# IL-17-Dependent Dysregulated Cutaneous Immune Homeostasis in the Absence of the Wiskott–Aldrich Syndrome Protein

**DOI:** 10.3389/fimmu.2022.817427

**Published:** 2022-02-21

**Authors:** Katherine E. Herman, Takeshi Yoshida, Angela Hughson, Alex Grier, Steven R. Gill, Lisa A. Beck, Deborah J. Fowell

**Affiliations:** ^1^ David H. Smith Center for Vaccine Biology and Immunology, Aab Institute of Biomedical Sciences, University of Rochester Medical Center, Rochester, NY, United States; ^2^ Department of Dermatology, University of Rochester Medical Center, Rochester, NY, United States; ^3^ Department of Microbiology and Immunology, University of Rochester Medical Center, Rochester, NY, United States; ^4^ Department of Microbiology and Immunology, Cornell University, Ithaca, NY, United States

**Keywords:** Wiskott–Aldrich syndrome, skin, immune homeostasis, IL-17, inflammation

## Abstract

Wiskott–Aldrich Syndrome (WAS) is characterized by recurrent infections, thrombocytopenia, and eczema. Here, we show that WASp-deficient mice on a BALB/c background have dysregulated cutaneous immune homeostasis with increased leukocyte accumulation in the skin, 1 week after birth. Increased cutaneous inflammation was associated with epithelial abnormalities, namely, altered keratinization, abnormal epidermal tight junctional morphology and increased trans-epidermal water loss; consistent with epidermal barrier dysfunction. Immune and physical barrier disruption was accompanied by progressive skin dysbiosis, highlighting the functional significance of the disrupted cutaneous homeostasis. Interestingly, the dysregulated immunity in the skin preceded the systemic elevation in IgE and lymphocytic infiltration of the colonic lamina propria associated with WASp deficiency. Mechanistically, the enhanced immune cell accumulation in the skin was lymphocyte dependent. Elevated levels of both Type 2 (IL-4, IL-5) and Type 17 (IL-17, IL-22, IL-23) cytokines were present in the skin, as well as the ‘itch’ factor IL-31. Unexpectedly, the canonical WAS-associated cytokine IL-4 did not play a role in the immune dysfunction. Instead, IL-17 was critical for skin immune infiltration and elevation of both Type 2 and Type 17 cytokines. Our findings reveal a previously unrecognized IL-17-dependent breakdown in immune homeostasis and cutaneous barrier integrity in the absence of WASp, targeting of which may provide new therapeutic possibilities for the treatment of skin pathologies in WAS patients.

## Introduction

Among patients with primary immunodeficiency disorders (PIDD), 40–70% have cutaneous pathology (1–4). Several PIDDs, including Wiskott–Aldrich Syndrome (WAS), present clinically with eczematous lesions (5). Over 70% of WAS patients develop severe, difficult-to-treat eczema which leads to significant morbidity, namely, disseminated cutaneous viral infections and fulminant sepsis (6, 7). The etiology of WAS eczema is unclear but the microbial complications are likely secondary to impaired anti-microbial immunity (8–10). In contrast to a defective IFN-γ response, patients with WAS deficiency develop robust type 2 responses, namely, elevated serum IgE, atopic-like eczema, and food allergy (11, 12).

Human WAS is caused by mutations in the *WAS* gene, a key regulator of actin cytoskeletal dynamics and gene transcription (13, 14). WASp expression, thought to be restricted to cells of the hematopoietic lineage, regulates many actin-dependent events such as leukocyte migration, adhesion, cellular polarization, and receptor signaling and also actin-independent transcriptional functions (15–20). In the absence of WASp, both innate and adaptive immunity is compromised with defects in phagocytosis, impaired CD8 T cell and NK cytotoxicity, reduced cytokine secretion, and altered migration (21, 22). Defects in regulatory function (Tregs, Bregs and anti-inflammatory, IL-10-producing, macrophages) also contribute to WAS pathologies resulting in unrestrained immune responses (11, 23–28).

Humans and mice lacking WASp display increased sensitization to food antigens (11) and develop poorly-restrained type 2 responses. Up to 10% of WAS patients and 100% of WASKO mice on the 129SvEv background develop inflammatory bowel disease (29). In WASp-deficient mice, the development of spontaneous colitis is dependent on both lymphocyte and innate cell subsets (22) and partially dependent on IL-4 (29). Defects in anti-inflammatory macrophages in WASp-deficient mice also lead to dysregulated intestinal homeostasis (28). Dysregulated immune cell trafficking may compromise immune homeostasis in the skin (30), with the failure of *WAS^−/−^* dendritic cells to migrate out of the skin following immune challenge leading to aberrant local immune activation and inflammation (31, 32). However, a spontaneous skin pathology in the WASp-deficient mouse similar to that of human WAS patients has not been characterized.

Here we show that WASp is necessary for the maintenance of skin barrier homeostasis. In BALB/c mice, the absence of WASp resulted in epidermal barrier disruption with tight junction aberrancies, increased transepidermal water loss (TEWL) and upregulation of the atopic dermatitis (AD) associated factor IL-31. This pathology was associated with innate and adaptive immune cell accumulation in the skin and a type 2- and type 17-skewed inflammatory milieu. Cutaneous immune dysfunction was abrogated in Rag^−/−^ *WAS* ^−/−^ mice demonstrating a lymphocyte-dependent dermatitis. Unexpectedly, IL-17, and not IL-4, was required for the immune dysfunction. Reduced cutaneous inflammation and reduced expression of both type 2 and type 17 cytokines in *WAS* ^−/−^ *IL-17* ^−/−^ mice suggests a critical role for type 17 inflammation in WAS-associated skin changes. These data show that WASp regulates the maintenance of epidermal barrier integrity and immune cell homeostasis in the skin through regulating homeostatic IL-17 responses.

## Methods

### Mice

Wild-type (WT) BALB/c were obtained from the NCI. *WAS* ^−/−^ C57BL/6 mice were provided by Dr. Janis Burkhardt (U. Penn). *WAS* ^−/−^ BALB/c were generated by backcross of *WAS* ^−/−^ C57BL/6 for at least 10 generations. IL17^−/−^ BALB/c mice were provided by Dr. Anna Valujskikh (Cleveland Clinic). IL-4^−/−^ BALB/c animals were purchased from Jackson Animal Laboratories. Rag2^−/−^ BALB/c mice were obtained from Dr. Terry Wright (U. Rochester). All animals were housed in the specific pathogen-free facility at the University of Rochester. All animal experimentation was reviewed and approved by the University of Rochester’s University Committee on Animal Resources and the Institutional Animal Care and Use Committee.

### Trans-Epidermal Water Loss

Fur on mouse flank skin was trimmed manually, then TEWL was measured using TM300 device from Courage-Khazaka Electronics (Cologne, Germany).

### Whole Mount Immunofluorescent Staining

For whole mount IF of epidermal sheets, hair was first removed from ears with depilatory cream (VEET). Ear dorsal and ventral sheets were separated and incubated in 0.5 M ammonium thiocyanate at 30°C for 20 min. Epidermis and dermis were separated and epidermal sheets fixed in 4% paraformaldehyde, washed and permeabilized with cold 100% methanol at −20°C. Epidermal sheets were blocked (1% BSA) and stained with polyclonal rabbit anti-claudin 1 Ab (Thermo Fisher; PAD : JAY.8) and species-specific AlexaFluor-tagged secondary Ab (Invitrogen) or AlexaFluor-tagged anti-mouse I-A/I-E (Biolegend: M5/114.15.2) and imaged using laser scanning confocal microscopy (Olympus FV1000). Three to four fields of view per epidermal sheet were captured and analyzed using Imaris v8.3.

### Flow Cytometry

Ears and flank skin were digested for 30 min at 37°C with collagenase/dispase. Colonic LP cells were isolated as previously described (33). Single cell suspensions were stained with a combination of the following antibodies: CD3 (BD; 145-2C11), CD4 (RM4-4, RM4-5), CD5 (53-7.3), CD8α (53-6.7), CD8β (53-5.8), CD11b (M1/70), CD11c (N418), CD45R/B220 (RA3-6B2), CD45 (30-F11), CD49b (Dx5), CD103 (2E7), CD127 (A7R34), CD205 (NLDC-145), CD207 (4C7), FcϵR1 (MAR-1), Siglec-F (E50-2440), Gr-1 (RB6-8C5), Ly6G (1A8), I-A/I-E (M5/114.15.2), T1/ST2 (DJ8), ICOS (C398.4A), NKp46 (29A1.4), KLRG1 (2F1), TER-119 (TER-119), γδ TCR(eBioGL3), Foxp3 (FJK-16s), GATA3 (L50-823), RORγt (Q31-378) and Live/Dead (Invitrogen). LSR-II flow cytometer acquisition and analysis using FlowJo software (Tree Star). Cell counts were determined by flow cytometry, from whole ear homogenates. The total homogenate from the ear pinna of each mouse was collected by flow cytometry and the total number of live, CD45^+^ cells calculated using 20,000 AccuCheck (Invitrogen) counting beads/sample for calibration, according to the manufacturers protocol.

### Cytokine Measurement

Ear tissue was digested in collagenase/dispase and homogenized in 500 μl PBS. Supernatants were assayed using the Milliplex Mouse Cytokine/Chemokine kits, per manufacturer’s instructions. Plates were read using Bio-Plex 200 instrument (Bio-Rad). For LN intracellular cytokine staining, 1 × 10^6^ LN cells in RPMI/10%FCS were stimulated at 37°C with PMA (Sigma; 50 ng/ml) and ionomycin (Calbiochem; 500 ng/ml) for 4 h. Brefeldin A (BD; 1 ug/ml) was added after 1 h of culture, for the remaining 3 h of culture. Cells were washed and surface stained of CD45 and CD4 before fixation and permeabilization (BD Cytofix/cytoperm). Cells were stained for intracellular cytokines IFNγ (eBioscience; XMG1.2), IL-4 (eBioscience; 11B11) and IL-17A (Biolegend; TC11-18H10.1) and analyzed using the LSR-II flow cytometer and FlowJo software.

### Serum IgE Measurement

Serum IgE was measured by ELISA.

### Skin Microbiome Analysis

Catch-All Sample Collection swabs (Epicentre) of mouse ears were stored in 2 ml Eppendorf Safe-Lock Biopur tubes containing 100 μl of Yeast cell lysis buffer (Epicentre) at −80°C. DNA was extracted using MasterPure Yeast DNA Purification Kit (Epicentre) and PureLink Genomic DNA Mini Kit (Thermo Fisher) and eluted with 40 μl MoBio PCR water (certified DNA-free). 16S rRNA V1-V3 amplicon libraries were sequenced using the Illumina HiSeq2500 Sequencer (San Diego, CA). Illumina reads were assessed for quality and then analyzed using phylogenetic and operational taxonomic unit methods in the Quantitative Insights into Microbial Ecology (QIIME) open source software v1.9.1.

### Statistical Analysis

Prism software (GraphPad) was used for all statistical tests except microbiome analyses. All data presented are mean ± SEM unless otherwise indicated. Statistical analysis of microbiome data was performed in R software. Alpha diversity: Shannon index and PD whole tree. Beta diversity: Bray–Curtis dissimilarity index and Unweighted UniFrac. Relative abundance of taxa were binned for WT vs. *WAS^−/−^*; p-values by Student’s t-test and 999 Monte Carlo permutations, adjusted for multiple comparison using the p.adjust function in R (method = ‘fdr’). *p <.05, **p <.01, ***p <.001, ****p <.0001, not significant (n.s.) p >.05.

## Results

### Spontaneous Skin Pathology and Epidermal Barrier Dysfunction in the Absence of WASp

**Figure 1:**
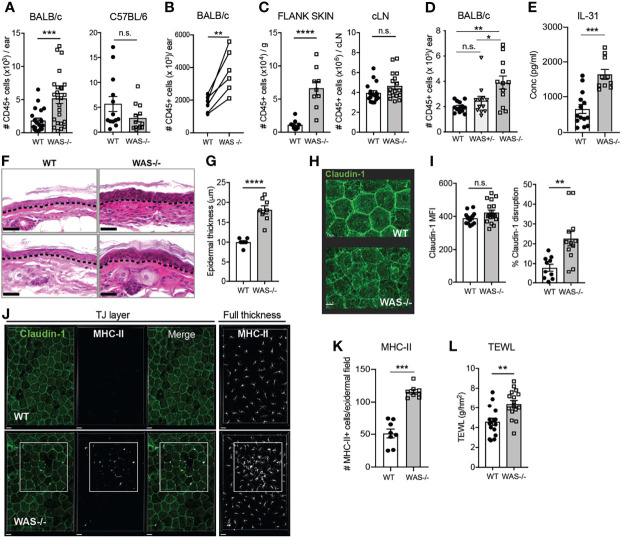
Spontaneous skin pathology and barrier dysfunction in WASp-deficient mice. **(A)** Number of CD45^+^ cells per ear pinna in 8-week BALB/c (left) and C57BL/6 (right) mice, measured by flow cytometry, combined data from 4 independent experiments. **(B)** Paired 8 week BALB/c WT and *WAS^−/−^* mice in individual experiments, each set of paired symbols represents the mean CD45^+^ cell count per ear pinna in a given experiment. **(C)** Number of CD45^+^ cells/g of dorsal flank skin (left) or cervical skin-draining LN of BALB/c mice. **(D)** Number of CD45^+^ cells per ear pinna WT, WAS^+/−^ and *WAS^−/−^* mice. **(E)** IL-31 measured by luminex assay from WT and *WAS^−/−^* BALB/c ear skin supernatants at eight weeks of age. **(A–E)**, n = 3–6/group, 2–6 independent experiments. **(F)** Hematoxylin and eosin-stained ear skin from WT BALB/c and *WAS^−/−^* mice. Dotted line, dermal–epidermal junction. Scale bar = 20 μm. **(G)** Epidermal thickness quantified from **(F)**, measured at thickest point. **(H)** Representative images of Claudin-1 expression in WT and *WAS^−/−^* BALB/c epidermal sheets, by confocal microscopy. Scale bar = 15 μm. **(I)** Quantification of total claudin-1 expression intensity from panel **(F)**. Right, quantification of claudin-1 disruption along length of epithelial cell border (see **Supplementary Figure 1**). **(J)** Representative images of MHC-II and claudin-1 staining in the TJ layer of epidermal sheets from WT and *WAS^−/−^* mice. Right panel, MHC-II staining in the full thickness epidermal sheet. **(K)** Quantitation of the number of MHC-II^+^ cells in each imaging field from panel **(J)**. Scale bar = 20 μm. **(J, K)** n = 3–4/group, 2 independent experiments. **(L)** TEWL measured from flank skin of WT and *WAS^−/−^* animals, n = 3–4/group, 4 independent experiments. All animals 8 weeks of age. *p <.05, **p <.01, ***p < .001, ****p <.0001, n.s., not significant p > .05, Student’s t-test.

We assessed homeostatic immunity in the ear skin of both adult (8-week old) C57BL/6 and BALB/c *WAS^−/−^* mice. No overt changes in immune cell numbers in the skin of *WAS^−/−^* C57BL/6 mice were found, however, *WAS^−/−^* BALB/c mice had significantly elevated numbers of CD45^+^ hematopoietic cells in the steady state (**Figure 1A**). We noted some variability between experiments in the overall magnitude of inflammation, but in all cases the difference between WT and *WAS^−/−^* mice was significant within each individual experiment (**Figure 1B**). Increases in CD45^+^ cells were also observed in the dorsal flank skin of *WAS^−/−^* BALB/c animals (**Figure 1C**, left panel). In contrast, no increase in CD45^+^ cells in the cervical skin-draining lymph node (LN) was observed, suggesting immune dysregulation was cutaneously, and not centrally, driven (**Figure 1C**, right panel). Moreover, cutaneous inflammation was only observed in the full absence of WASp, WAS^+/−^ mice showing no overt skin inflammation (**Figure 1D**). Accompanying the increase in leukocyte infiltration was the upregulation of the atopic dermatitis-associated pruritogenic ‘itch’ factor IL-31 (**Figure 1E**).

To determine if the elevated immune cell accumulation in the skin had physiological effects on the skin barrier, we first examined histological sections of mouse ear skin. At eight weeks of age, we observed dysmorphology of *WAS^−/−^* mouse skin architecture, with parakeratosis and significant thickening of the epidermis compared to WT (**Figures 1F, G**). Parakeratosis may indicate altered keratinocyte differentiation and changes to the epidermal tight junction (TJ) network (34–38). We used confocal microscopy to visualize expression patterns of claudin-1 deposition, a major skin TJ protein, in epidermal sheets prepared from WT and *WAS^−/−^* mouse skin (**Figure 1H**). The WT epidermis showed clear claudin-1 stain localized at interepithelial junctions in a typical honeycomb pattern (39), while the epidermis from WASp-deficient mice showed regions of claudin-1 staining with poorly defined intercellular borders. There were no differences in total claudin-1 epidermal expression between WT and *WAS^−/−^* skin (MFI) (**Figure 1I**, left panel), but quantification of the contiguous intensity of claudin-1 staining along the epithelial cell borders revealed *WAS^−/−^* claudin-1 expression was significantly more irregular than WT (**Figure 1I**, right panel; **Supplementary Figure 1**). In response to TJ disruption, Langerhan cells (LCs) have been reported to extend dendrites out into the TJ area to ‘plug’ holes in a disrupted epidermis. Consistent with a TJ breach, we observed an increase in MHC Class II^+^ projections (predominantly from CD11b^+^CD11c^+^Langerin^+^ cells) between epithelial cells in *WAS^−/−^* epidermal sheets (**Figure 1J**, TJ layer panels). Moreover, there was a marked accumulation of MHC-II^+^ cells in the epidermal sheets (**Figure 1J**, right panel; **Figure 1K**), as previously noted (31, 32).

These data suggest a disruption in the interface between the physical and immune barriers of the skin in the absence of WASp. Disrupted tight junctions in the epidermis in human atopic dermatitis corresponds to alterations in skin permeability to water (37, 39). *WAS^−/−^* mice had significantly elevated trans-epidermal water loss (TEWL) values compared to WT mice, indicating increased skin permeability and epidermal barrier dysfunction (**Figure 1L**). Thus, we find loss of WASp leads to both cutaneous immune dysregulation and skin barrier disruption.

### Enhanced Immune Cell Accumulation in the Skin of Mice Lacking WASp

**Figure 2:**
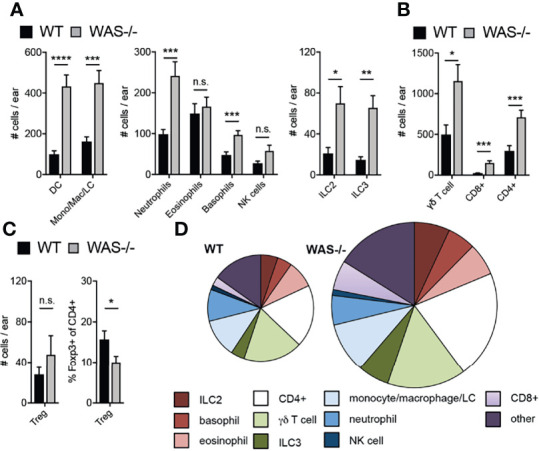
Accumulation of immune cells in skin of mice lacking WASp. **(A–C)** Number of immune cell subsets in the skin of the ear pinna, assessed by multiparameter flow cytometry. **(D)** Pie graphs represent relative frequencies of immune subsets (% of CD45^+^) in WT (left) and *WAS^−/−^* skin of the ear pinna (right). Size of pie represents relative number of CD45^+^ cells. All mice 8 weeks of age. n = 3–6/group, 2–5 independent experiments. *p <.05, **p <.01, ***p <.001, ****p <.0001, n.s., not significant p > .05 Student’s t-test.

Immune composition by flow cytometry in the skin at eight weeks of age revealed a general increase in many immune cell subsets in *WAS^−/−^* mice (subsets defined in **Supplementary Table 1**). Most innate cell subsets tested were enhanced including neutrophils, basophils, CD11c^+^ dendritic cells (DCs), LCs, NK cells, and CD11b^+^ monocytes/macrophages (**Figure 2A**) and increases in group 2 and 3 innate lymphoid cells (ILC2 and ILC3, **Figure 2A**). Eosinophils were, notably, not elevated in the absence of WASp (**Figure 2A**). CD4^+^ T cells, CD8^+^ T cells, and γδ TCR^+^ T cells were also significantly increased in *WAS^−/−^* skin (**Figure 2B**). Foxp3^+^ CD4+ T regulatory cell (Treg) numbers were only modestly enhanced in *WAS^−/−^* skin (**Figure 2C**, left panel) resulting in a significant reduction in the proportion of Tregs among CD4^+^ T cells (**Figure 2C**, right panel). Aside from Tregs, we found no significant alterations in the relative frequencies of the elevated immune cell subsets we evaluated (**Figure 2D** and **Supplementary Figure 2**). Thus, we observed significant increases in multiple skin immune cell populations, indicating broad dysregulation of skin immunity in WASp-deficient mice.

### Early Accumulation of Immune Cells in Skin of Mice Lacking WASp

**Figure 3:**
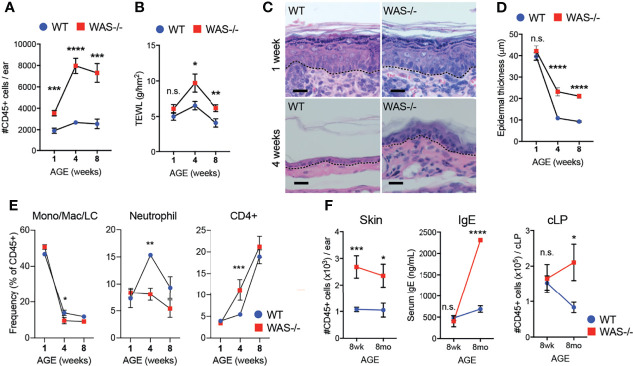
Immune cell accumulation precedes development of barrier dysfunction in mice lacking WASp. **(A)** Number of CD45^+^ cells per ear in mice aged 1, 4, and 8 weeks. **(B)** TEWL measured from flank skin of WT and *WAS^−/−^* animals from panel **(A)**. **(C)** Hematoxylin and eosin-stained ear skin from 1 and 4 week-old WT BALB/c and *WAS^−/−^* mice. Dotted line denotes dermal–epidermal junction. Scale bar = 20 μm. **(D)** Epidermal thickness quantified from ear skin sections in panel **(C)**, measured at thickest point. **(A–D)** n = 3–5/group, 2 experiments. **(E)** Frequencies of immune subsets at 1, 4, and 8 weeks of age in WT and *WAS^−/−^* mice. **(F)** CD45^+^ cellular infiltrate in the skin (left), serum IgE (middle) and CD45^+^ cellular infiltrate in the colonic lamina propria (cLP) (right) of mice aged 8–10 weeks and 8 months. n = 3–5/genotype/age group, 4 independent experiments. *p <.05, **p <.01, ***p <.001, ****p <.0001, n.s., not significant p > .05 Student’s t-test.

Immune cell cytokines could induce the observed epidermal barrier defects (40–43), or early barrier disruption could enhance immune cell accumulation. To gain mechanistic insight into the skin pathology, we assessed the concordance of immunologic changes and epidermal barrier dysfunction in the skin of WASp-deficient mice kinetically starting at 1 week of age. We detected increased CD45^+^ cell infiltration in *WAS^−/−^* skin as early as one week of age (**Figure 3A**). At this time, a modest, but not significant, increase in TEWL was observed in *WAS^−/−^* mice (**Figure 3B**) but no change was seen in the young (1 wk) skin architecture between WT and *WAS^−/−^* mice (**Figure 3C**). In contrast, physical barrier dysfunctions (TEWL, epidermal thickness) were significantly elevated by 4 weeks of age (**Figures 3B, D**) at the height of immune accumulation (**Figure 3A**). Thus, altered immune cell accumulation appears to precede the epidermal skin barrier dysfunction.

With respect to immune cells, neonatal WT and *WAS^−/−^* had a similar balance of immune cells, heavily skewed to monocyte/macrophage populations (**Figure 3E**). However, at four weeks of age, *WAS^−/−^* mice showed an enhanced frequency of CD4^+^ cells in the skin, with a concomitant decrease in the proportion of neutrophils and monocytes/macrophages present in the skin (**Figure 3E**). Interestingly, these early defects in skin immunity came weeks before the WASp-associated elevation in serum IgE and before lymphocytic infiltration of the colonic lamina propria (**Figure 3F**).

### Skin Dysbiosis in Mice Lacking WASp

**Figure 4:**
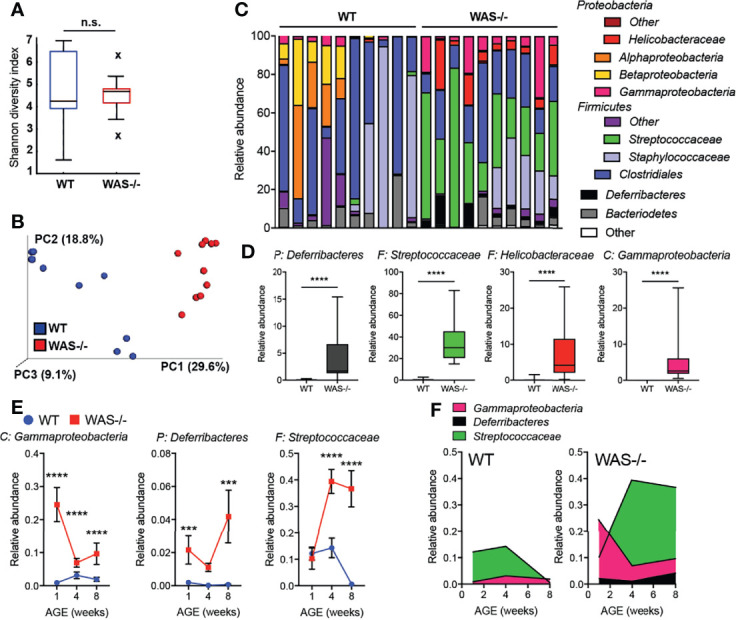
Dysbiosis in *WAS^−/−^* mouse skin. **(A)** Shannon diversity index of WT and *WAS^−/−^* skin microbiome samples collected at 8 weeks of age. **(B)** Principle coordinate analysis generated using unweighted UniFrac matrix. **(C)** Relative abundances of 12 major phyla/class/family taxonomies in 10 WT and 10 *WAS^−/−^* microbiome samples. **(D)** Taxa significantly enhanced in *WAS^−/−^* skin over WT by multivariate analysis (p <.05, beta coefficient >.1). P, phylum, C, class, F, family. **(E)** Kinetics of changes in *WAS^−/−^* signature microbial taxa in WT and *WAS^−/−^* animals at 1, 4, and 8 weeks of age. **(F)** Relative abundance of selected taxa in panel **(E)**. One-way ANOVA with Tukey’s post-test, ***p <.001, ****p <.0001, n.s., not significant p > .05.

Significant alterations in the skin microbiome have been reported in humans with primary immunodeficiencies and in AD (44–46). To investigate whether the immune and barrier dysregulation in the *WAS^−/−^* skin impacted the skin microbiome, we performed 16S ribosomal RNA (rRNA) sequencing on skin swabs from ear skin. Using the Illumina MiSeq platform, we obtained an average of 143,474 ± 40,444 V1–V3 reads/sample for 10 WT and 10 *WAS^−/−^* mice at eight weeks of age. Rarefaction curves indicated good coverage for dominant species present in the skin (**Supplementary Figure 3**). We detected no significant differences in alpha diversity between WT and *WAS^−/−^* (WT mean: 4.7 ± 1.8, *WAS^−/−^* mean: 4.5 ± 0.9 p = .773) using the Shannon diversity index (which assesses microbial community richness and evenness within a single sample) (**Figure 4A**). However, we observed significant clustering of *WAS^−/−^* samples separate from WT using the unweighted Unifrac method and principle coordinate analysis for assessing beta diversity (**Figure 4B**) (p = 1.41E−18). Changes in relative abundance of a number of bacterial communities was observed with WASp-deficiency (**Figure 4C**) with enrichment of the *Streptococcus* and *Helicobacter* genera, and also the *Deferribacteres* phylum (**Figure 4D**). Furthermore, there was colonization of a particular *Gammaproteobacteria* species, *Aggregatibacter pneumotropica*, on *WAS^−/−^* mouse skin that was not detected on WT skin (**Figure 4D**).

To assess the concordance of dysbiosis with the onset of skin pathology, we analyzed the skin microbiome at 1, 4, and 8 weeks of age (**Figures 4E, F** and **Supplemental Figure 4**). At one week of age, a striking change in the representation of microbial taxa in *WAS^−/−^* mice was already evident (**Figures 4E, F**). Elevations in *Gammaproteobacteria* and members of the *Deferribacteres* phyla at 1 week, remained elevated above WT at all timepoints (**Figures 4E, F**). The relative abundance of *Streptococcus* spp. in the *WAS^−/−^* mice was not seen as a neonate but increased in representation with age (**Figures 4E, F**), possibly as a secondary consequence of cutaneous inflammation. Therefore, the dysbiosis in *WAS^−/−^* mice appears coincident with the early immune dysfunction in the skin of WASp-deficient mice. Preliminary co-housing experiments suggest that the altered microbiome of *WAS^−/−^* skin is not sufficient to induce skin inflammation in WAS-sufficient (WT) mice (**Supplementary Figure 5**). Four week old WT and *WAS^−/−^* mice were co-housed for two weeks and then assessed for ear skin inflammation. After two weeks of co-housing, WT and *WAS^−/−^* skin microbiomes had equilibrated, containing a similar microbial profile (**Supplementary Figure 5**). This microbial mixing was mainly attributed to the acquisition of elevated WASp-associated *Gammaproteobacteria*, *Deferribacteres*, and *Streptococcaceae* by WT mice, with no significant impact of the WT microbiome on the co-housed WAS*^−/−^* mice. However, we observed no induction of skin inflammation associated with these changes in the skin microbiome in WT cohoused mice (**Supplementary Figure 5**), suggesting that the microbiome of *WAS^−/−^* mice is not sufficient to drive cutaneous inflammation in immunocompetent WT hosts.

### Type 2- and Type 17-Biased Inflammatory Milieu in Skin of Mice Lacking WASp

**Figure 5:**
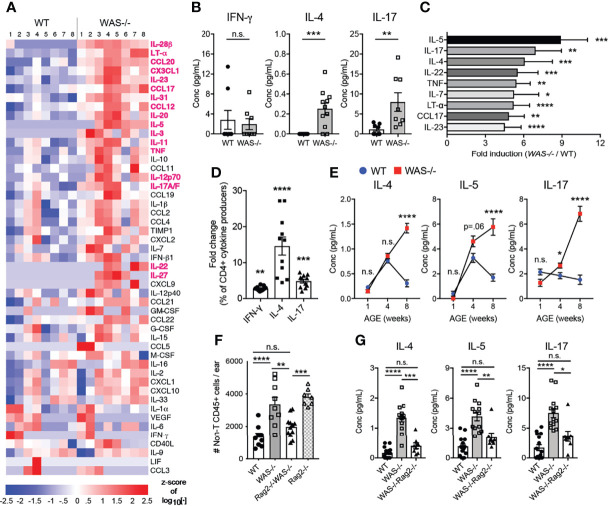
Biased type 2 and 17 inflammation in *WAS^−/−^* skin at eight weeks of age requires adaptive immune cells for development. **(A)** Heat map of Z-score of analytes measured by luminex assay from WT and *WAS^-/-^* BALB/c ear skin supernatants. Factors in magenta p <0.01. **(B)** Absolute values of cytokines measured by luminex as in panel **(A)**. **(C)** All factors with fold change greater than four (*WAS^−/−^*:WT). **(A–C)** n = 8–11/group, 2 independent experiments. **(D)** Cytokine frequencies among CD4^+^ cells from the skin-draining LN following PMA/ionomycin stimulation and intracellular cytokine staining, fold change (*WAS^−/−^*:WT) n = 3–5/group, 3 independent experiments. **(E)** Cytokines in ear skin homogenates at 1, 4, and 8 weeks of age. **(A–D)** All mice 8 weeks of age. Statistics: Mann–Whitney U test. Statistics for **(E)**: One-way ANOVA with Tukey’s post test. *p <.05, **p <.01, ***p <.001, ****p <.0001. **(F)** Total CD45^+^CD4^−^CD8β^−^γδ^−^ cells in ears of WT, *WAS^−/−^*, *WAS^−/−^Rag2^−/−^* and *Rag2^−/−^* mice. **(G)** Absolute values of cytokines measured by Luminex. All mice 8 weeks of age, n = 2–5/group, 4 independent experiments. Statistics **(F, G)** One-way ANOVA with Tukey’s post test. *p <.05, **p <.01, ***p <.001, ****p <.0001, n.s., not significant p > .05.

We next measured inflammatory mediators in the mouse ear skin. We found marked differences in cytokines and chemokines in the skin of *WAS^−/−^* mice using a multiplex Luminex assay (**Figure 5A**). Out of 55 analytes tested (**Supplementary Table 2**), 18 analytes were significantly upregulated in *WAS^−/−^* versus WT skin (**Figure 5A**, highlighted in magenta) and no factors were downregulated. IL-4 and IL-17 were both significantly enhanced in the *WAS^−/−^* skin, but there was no increase in IFN-γ, suggesting a bias toward type 2 and/or 17 responses (**Figure 5B**). Indeed, type 2 cytokines (IL-4, IL-5) and type 17 cytokines (IL-17, TNF, IL-22, IL-23) were among the most upregulated analytes (**Figure 5C**). CCL17, whose cognate receptor CCR4 is expressed by Th2/Th17 CD4^+^ cells, was particularly enriched in *WAS^−/−^* skin (**Figure 5C**). This chemoattractant cue may promote the preferential accumulation of type 2/17 effectors in the skin (47) along with the AD-associated pruritogenic cytokine IL-31 (**Figure 1C**) (48). To determine whether the type 2/17 inflammatory bias present in the skin was associated with changes in LN Th differentiation, we assessed cytokine production by CD4^+^ T cells from the skin-draining LN. Upon *in vitro* re-stimulation, IL-4 was upregulated 14.62 fold, IL-17 by 4.887 fold and IFN-γ by 2.853 fold in *WAS^−/−^* CD4^+^ T cells compared to WT (**Figure 5D**). Thus, the type 2 cytokine enrichment (and to a lesser extent type 17) we observed in the skin may be associated with enhanced priming or potential for IL-4/IL-17 production in the skin draining LN. In the skin, γδ T cells were also sources of elevated IL-17 (data not shown). Kinetically, the elevated inflammatory cytokines in the skin at 8 weeks (**Figure 5C**), were not enhanced at 1 week of age. However, IL-5 and IL-17 cytokines were upregulated in the *WAS^−/−^* skin by 4 weeks of age (**Figure 5E**).

To assess the role of the adaptive immune compartment in the cutaneous immune dysregulation, we crossed *WAS^−/−^* mice to *Rag2^−/−^* mice on the BALB/c background (*WAS^−/−^Rag2^−/−^*). Loss of T and B cells abolished WAS-associated skin inflammation, as determined by normalizing for the non-T cell CD45^+^ cell (CD45^+^CD4^−^CD8β^−^γδ^−^) compartment between *WAS^−/−^* and *WAS^−/−^Rag2^−/−^* (**Figure 5F**). The results suggest the adaptive immune compartment actively drives the enhanced innate cell recruitment in *WAS^−/−^* skin. However, interpretation of these experiments is complicated by the presence of an unexpected skin inflammation in the *Rag2^−/−^* mice themselves. Loss of both T cells and WASp, *WAS^−/−^Rag2^−/−^*, led to the amelioration of inflammation. At the cytokine level, elevated cytokine production in the *WAS^−/−^* skin was reduced to WT levels in the *WAS^−/−^Rag2^−/−^* mice (**Figure 5G** and **Supplementary Table 3**). Thus, lymphocytes appear to be essential for the initiation and/or amplification of cutaneous pathology in WASp-deficient mice.

### IL-17, But Not IL-4, Contributes to the Immune Pathology in Mice Lacking WASp

**Figure 6:**
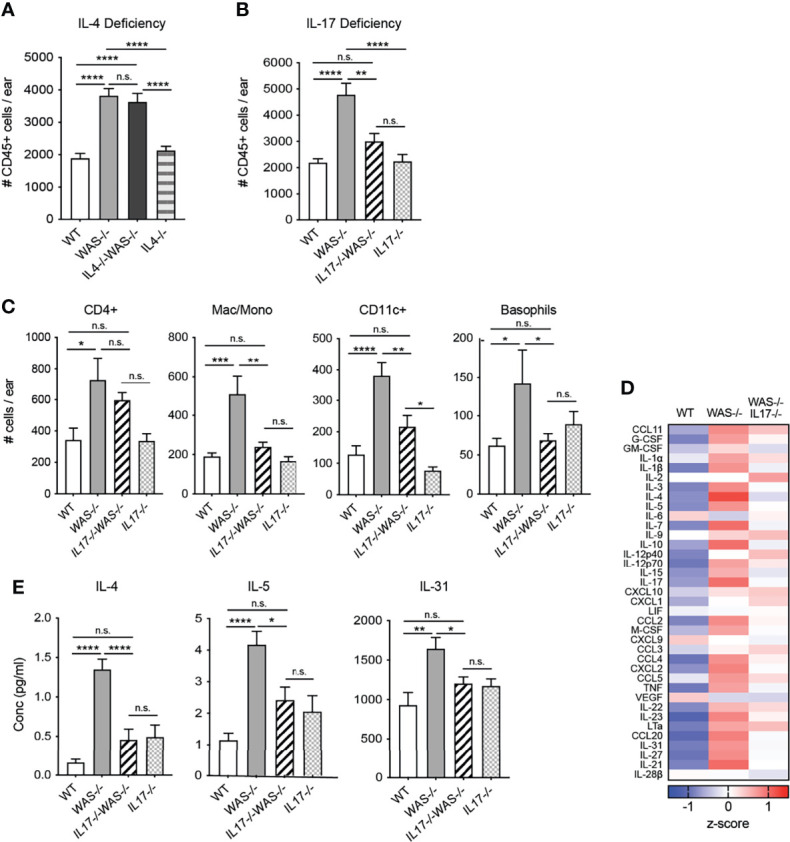
IL-17, but not IL-4, necessary for the development and/or maintenance of skin inflammation in mice lacking WASp. **(A)** Total CD45^+^ cells in ears of WT, *WAS^−/−^* and *WAS^−/−^ IL-4^−/−^* and *IL-4^−/−^* BALB/c mice. **(B)** Total CD45^+^ cells in ears of WT, *WAS^−/−^* and *WAS^−/−^ IL-17^−/−^* and *IL-17^−/−^* BALB/c mice. **(A, B)** Total CD45^+^ cells in ear measured by flow cytometry. **(C)** Number of immune cell subsets in ear skin. **(D)** Heat map of Z-scores of analytes measured by luminex assay from ear skin supernatants. **(E)** Absolute values of cytokines measured by Luminex assay as in panel **(E)**. n = 3–5/genotype, 3 independent experiments. *p <.05, **p <.01, ***p <.001, ****p <.0001, n.s., not significant p > .05, One-way ANOVA with Tukey’s post test. All mice at 8 weeks of age.

In the gut, elevated IL-4 has been previously associated with enhanced serum IgE and colitis in WASp-deficient mice (29). Recent studies in an adoptive transfer system of Th1/Th17 colitis have shown elevated IFNγ and IL-17 in the gut when macrophages lack WASp, but the functional significance of IL-17 was not explored (28, 29). Therefore, we assessed the role of IL-4 and IL-17 in the development of skin inflammation using *WAS^−/−^IL-4* ***^-/-^*** and *WAS^−/−^IL-17^−/−^* mice (**Figures 6A, B**). IL-4 deficiency failed to alleviate the dysregulated accumulation of immune cells in the *WAS^−/−^* skin (**Figure 6A**). Furthermore, we found little change in composition of immune cell subsets or the cytokine/chemokine milieu in skin of *WAS^−/−^IL-4^−/−^* compared to *WAS^−/−^* mice (**Supplementary Figure 6**). In contrast, we found that total CD45^+^ cells were significantly decreased in WASp-deficient skin in the absence of IL-17 (**Figure 6B**). Lack of IL-17A in WASp-deficient animals led to significant reductions in the accumulation of innate cell types such as monocytes/macrophages/LCs, basophils and CD11c^+^ dendritic cells (**Figure 6C**), suggesting that IL-17A may play a role in the tissue recruitment or expansion of these populations in *WAS^−/−^* animals. CD4+ T cells counts trended down in *WAS^−/−^* groups in the absence of IL-17, but the *WAS^−/−^IL-17^−/−^* phenotype was intermediate between WT and *WAS^−/−^* groups, with no significant differences when compared to either group.

Most notably, loss of IL-17 was accompanied by significant reductions in many of the inflammatory mediators found elevated in the *WAS^−/−^* skin (**Figure 6D**), to levels similar to that of immunocompetent WT and *IL-17^−/−^* mice. In particular, we found a striking reduction in the type 2 cytokines IL-4 and IL-5 in the *WAS^−/−^IL-17^−/−^* skin compared to *WAS^−/−^* controls (**Figure 6E**). The pruritogenic factor IL-31 was also significantly reduced to WT levels in the skin of *WAS^−/−^IL-17^−/−^* mice (**Figure 6E**). Our observations point to IL-17A, but not IL-4, contributing to the development and/or maintenance of the skin inflammation described in *WAS^−/−^* animals. Interestingly, as noted in other murine models of asthma and eczema (49–51), IL-17 appears to support or amplify type 2 cytokines in WASp-deficient animals. Our results uncover a previously unappreciated spontaneous breakdown in cutaneous immune homeostasis in the absence of WASp driven by elevated IL-17. Immune dysregulation occurred soon after birth and was associated with altered skin TJ morphology and barrier function resulting in dysbiosis.

## Discussion

Despite the prominence of eczema in human WAS patients, little is known about the pathogenesis of skin inflammation. This may be due in part to a lack of a cutaneous pathological phenotype in WASp-deficient mice. While accumulation of Langerhans cells in the steady-state epidermis has been previously observed, to our knowledge no overt changes in total immune cell number or changes in skin architecture have been reported (32). In this study, we showed that integrating the WASp deficiency into the BALB/c background led to the development of a spontaneous dermatitis associated with a type 2 and type 17 inflammatory milieu, significant changes in epidermal morphology and barrier dysfunction (see model, **Supplementary Figure 7**). This immune and physical barrier dysfunction was concomitant with microbial dysbiosis. IL-17, and not IL-4, was a key driver of the immune inflammation in the skin.

From a very young age, mice lacking WASp displayed a marked enrichment of immune cells in the skin. While we do not yet know the inciting factor, a number of studies in *WAS^−/−^* mice have demonstrated impaired LC and DC trafficking out of the skin in response to cutaneous sensitization, suggesting the early leukocyte accumulation may arise due to initial defects in immune cell egress from the skin (31, 32, 52). Aberrant immune cell accumulation in the skin was associated with marked changes in physical epidermal organization and barrier function. Inflammatory cytokines are known to reduce skin barrier function by modulating keratinocyte expression of tight junctional proteins and altering keratinocyte maturation/differentiation (40–42, 53). These physical changes may alter the topography of niche environments available to skin microbiota and/or enhance commensal translocation and access to the immune system (54). Our findings suggest that in the absence of WASp, Langerhan cells are distinctly positioned to respond to environmental antigens. Enhanced contact with microbial stimuli may amplify the homeostatic disruption leading to the overexpression of the type 2 and type 17 cytokines/chemokines. Reduced immunosuppression (26) with WASp-deficiency likely conspires to propagate the inflammatory response in the skin.

The resident skin immune system tunes responses to commensals to maintain barrier immunity and protection without overt inflammation (55). In turn, commensal organisms have been associated both with initiation of, and protection from, mucosal pathology (56–58). Similar to our results in the skin, *Helicobacter* spp. was strongly associated with the development of inflammatory colitis in 129SvEv *WAS^−/−^* mice (59). The presence of *Streptococcus* in the *WAS^−/−^* mice is also seen in AD-prone skin (60). In contrast, *Gammaproteobacteria* have been linked to protection from allergy through IL-10 induction (61). Our studies showed dominant *Gammaproteobacteria* early in *WAS^−/−^* skin was rapidly replaced by an abundance of *Streptococcus*. Whether dysbiosis elicits or is a consequence of barrier dysfunction is a long-outstanding question in AD. Preliminary studies co-housing WT mice in the same microbial environment as the *WAS^−/−^* BALB/c mice was not sufficient to induce skin inflammation (**Supplementary Figure 5**). Moreover, pilot experiments with transfer of the BALB/c *WAS^−/−^* skin microbiome to WT germ-free mice also failed, by itself, to promote skin inflammation. Our results suggest that WAS-mediated cutaneous immune dysregulation may initially occur independently of microbial cues.

The combination of WAS-deficiency and the BALB/c genetic background was necessary to reveal dysregulated cutaneous immunity. There are significant differences between C57BL/6 and BALB/c strains including major histocompatibility complex (MHC) haplotype and type 1 and type 2 cytokine biases (62, 63). Given the predisposition of BALB/c animals to develop type 2 responses, it is possible that genetic background strain polymorphisms (at non-WAS loci) associated with heightened type 2 immunity synergize with WASp-deficiency to precipitate overt type 2 and type17 cutaneous inflammation. Our data suggest that in human WAS, additional genetic factors may contribute to differences in disease heterogeneity or severity.

Perhaps our most provocative observation is that cutaneous dysregulation preceded both LP inflammation and serum IgE elevation. Disrupted skin barrier in AD is thought to contribute to the development of food allergy and asthma through epicutaneous sensitization to environmental antigens (64–66). Indeed, in WASp-deficient mice many of the circulating IgE antibodies appear to be specific for chow components (11). Cutaneous exposure to food and environmental antigens *via* disrupted eczematous skin is thought to reduce tolerance to these antigens and lead to the development of specific IgE (67). Cutaneous sensitization is the major hypothesis behind the “atopic march” in patients with AD (68–72): the progression of eczema in infancy to food allergy and asthma/allergic rhinitis later in life (73). The spontaneous early skin immune dysregulation, in addition to food allergy and serum IgE elevation, in the *WAS^−/−^* mouse model may be a useful tool for understanding the evolution of atopy and determining ways to halt its progression.

Notably, the spontaneous dermatitis in WASp-deficient mice was dependent on IL-17 and not IL-4. Loss of IL-17 also abrogated the dysregulated IL-4 response. At this stage, we do not know if the elevated IL-17 directly drives the elevated Type 2 cytokines or whether it contributes indirectly by disrupting skin homeostasis and facilitating aberrant Type 2 responses. IL-17A can induce Th2 responses in murine models of atopic dermatitis and Th2 cells making both IL-4 and IL-17 have been seen in human allergic asthma and AD and may correlate with disease activity (74–76). Interestingly, recent work suggests that IL-17/IL-22 responses are particularly elevated in pediatric atopic dermatitis (77, 78). While a direct comparison of our mouse studies to human WAS is not possible without further investigation of the cutaneous pathology in humans, assessment of IL-17 in the difficult-to-treat eczema experienced in WAS patients could provide a new pathway for targeted treatment to alleviate the cutaneous disease.

## Data Availability Statement

The datasets presented in this study can be found in online repositories. The names of the repository/repositories and accession number(s) can be found below: NCBI, PRJNA790974.

## Ethics Statement

All mice were maintained in a pathogen-free facility at the University of Rochester Medical Center. All mouse procedures were performed with approval of the University of Rochester’s Institutional Animal Care and Use Committee.

## Author Contributions

KH designed and conducted experiments, analyzed the data and wrote the manuscript. TY conducted experiments and analyzed the data. AH conducted experiments and analyzed the data. LB helped design the experiments, provided expertise/reagents and edited the manuscript. AG analyzed the microbiome data. SG helped design the experiments, provided expertise/reagents and edited the manuscript. DF designed the experiments, analyzed the data and wrote the manuscript. All authors listed have made a substantial, direct, and intellectual contribution to the work and approved it for publication.

## Funding

This work was supported by grants from the NIH NIAID: AI072690 and AI102851 awarded to DF and the T32 AI118689 to KH.

## Conflict of Interest

The authors declare that the research was conducted in the absence of any commercial or financial relationships that could be construed as a potential conflict of interest.

## Publisher’s Note

All claims expressed in this article are solely those of the authors and do not necessarily represent those of their affiliated organizations, or those of the publisher, the editors and the reviewers. Any product that may be evaluated in this article, or claim that may be made by its manufacturer, is not guaranteed or endorsed by the publisher.
