# Supplementary material for: IL-17-Dependent Dysregulated Cutaneous Immune Homeostasis in the Absence of the Wiskott–Aldrich Syndrome Protein
Source: Front Immunol. 2022 Feb 21;13:817427. doi: 10.3389/fimmu.2022.817427 (PMC8900519; doi:10.3389/fimmu.2022.817427)
Supplement: Supplementary file 1 [file DataSheet_1.pdf]

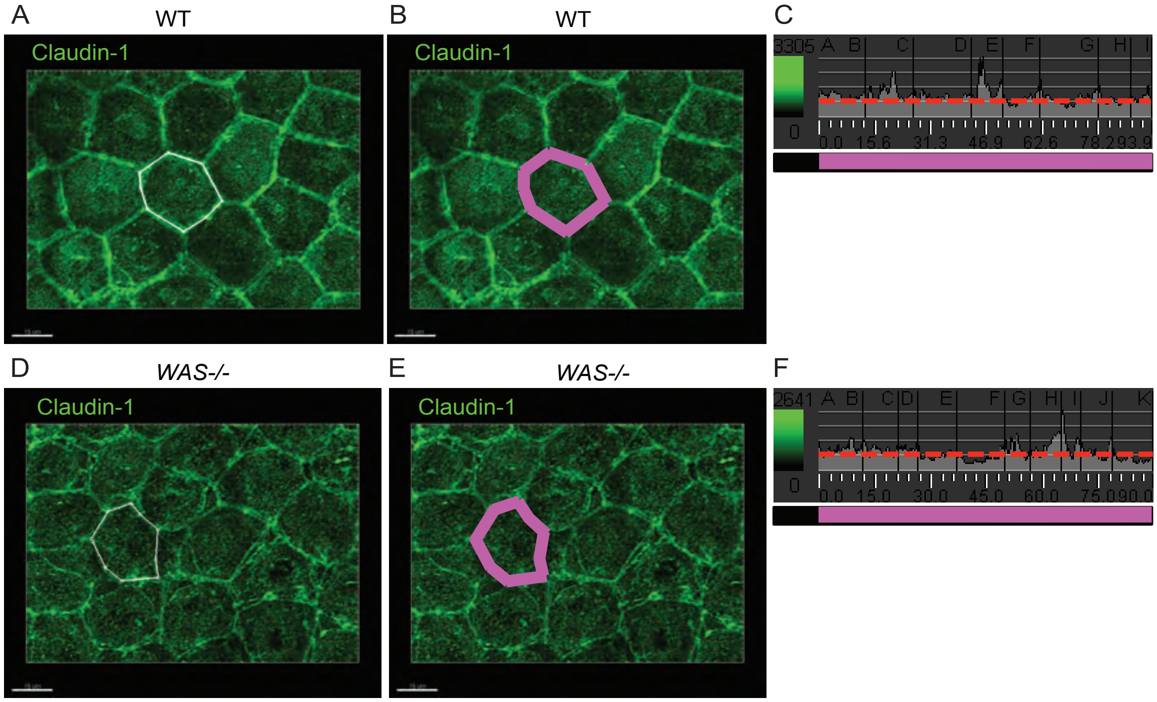

Supplemental Figure 1: Quantification of claudin-1 expression in outer epidermis. (A,D): Confocal image of Claudin-1 expression in WT (A) and WAS -/- (D) epidermis. (B, E): Interepithelial cell border outlined in magenta in WT (B) and WAS -/- (E) epidermis. (C, F): Histogram of claudin-1 expression quantified along interepithelial cell border (magenta). Red dashed line denotes lowest quartile of claudin-1 expression. To calculate claudin-1 loss in Fig 1G, the length of border with claudin-1 expression below this threshold was divided by the total border length.

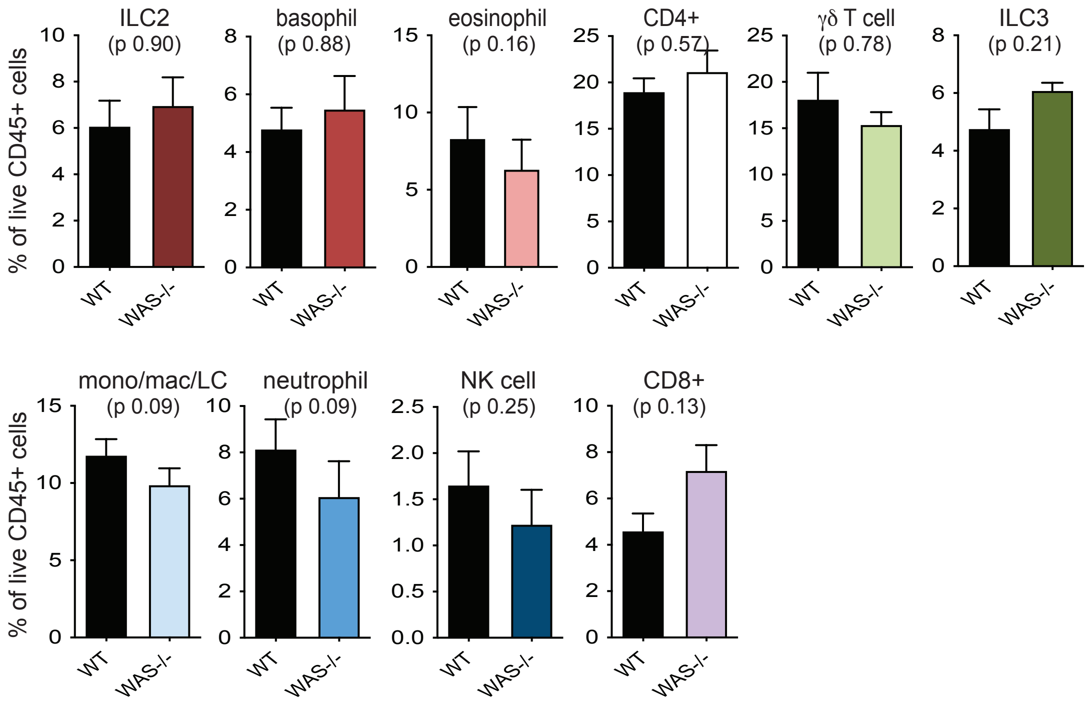

Supplemental Figure 2: Frequency of immune cell subsets in WT and WAS-/- skin. Frequency of each immune cell subsets within the live CD45+ cell population. Pooled data from n=4-6/genotype, 3 independent experiments. Not significant: p >0.05, Mann Whitney. mono/mac/LC: moncyte/macrophage/LC. Combined data from 4 independent experiments.

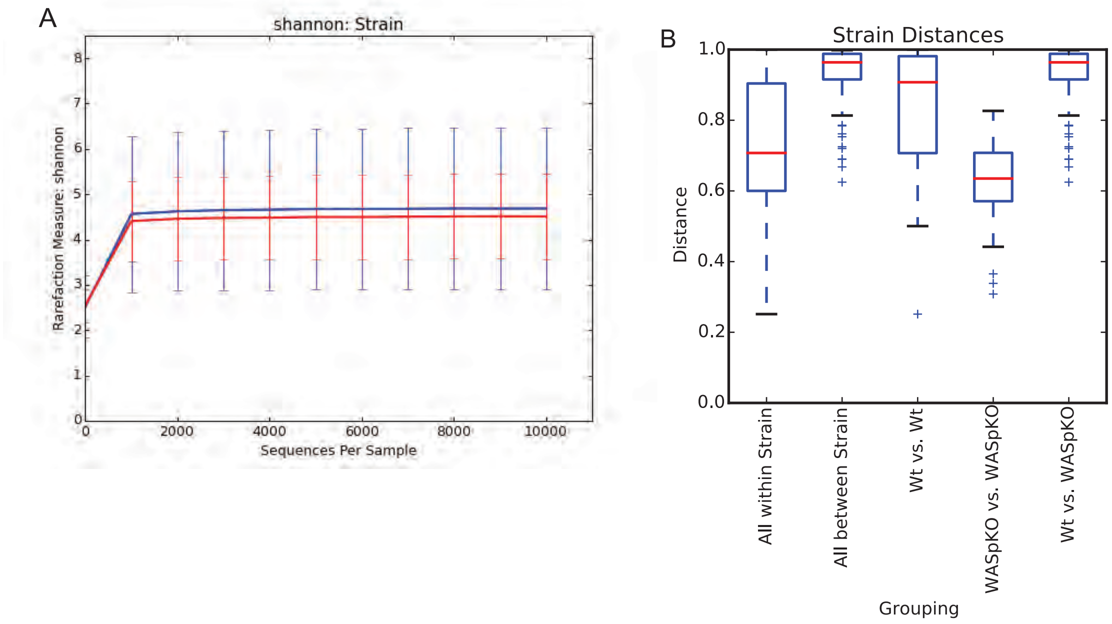

Supplemental Figure 3: Microbiome rarefaction curves and strain distance calculations
(A) Shannon diversity index of WT and WAS -/- skin microbiome samples. Rarefaction analysis (left). Curve represents mean of each strain +/- SD over 10000 sequences. (B) B: Strain distances summary calculated by Bray-Curtis PCA.

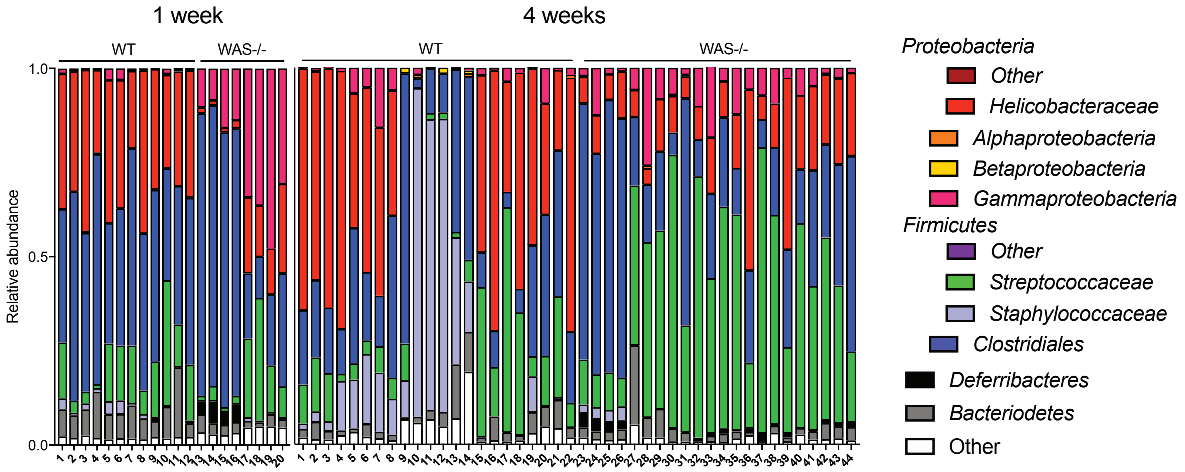

Supplemental Figure 4: Microbiome taxa relative abundance at 1 and 4 weeks. Bacterial taxonomic classifications show unique representation of taxa in skin microbiome of WT and WAS -/- mice at both one and four weeks of age. Relative abundances of 12 major phyla/class/order taxonomies in 12 WT and 8 WAS -/- microbiome samples at one week of age (left) and 22 WT and 22 WAS -/- microbiome samples at four weeks of age (right).

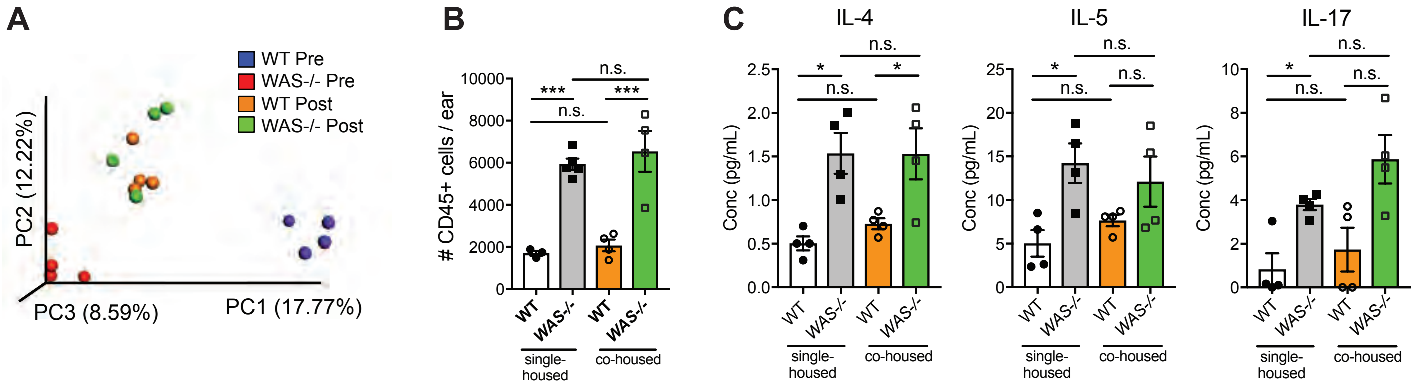

Supplemental Figure 5: Co-housing effect on microbiome and skin inflammation. (A) Skin microbiome of WT and *WAS*-/- animals was collected prior to (pre-) or after (post-) 2 weeks of co-housing, beginning at 4 weeks of age. Principle coordinate analysis generated using unweighted UniFrac matrix of WT and *WAS*-/- skin microbiome samples. (B) Total CD45+ cells present in the ear of single-housed or co-housed animals, quantified by flow cytometry. (C) Absolute values of cytokines in ear skin supernatants of single-housed and co-housed animals, measured by luminex. n=4/group, 1 independent experiment. One-way ANOVA with Tukey's post-test. *p<.05, ***p<.001.

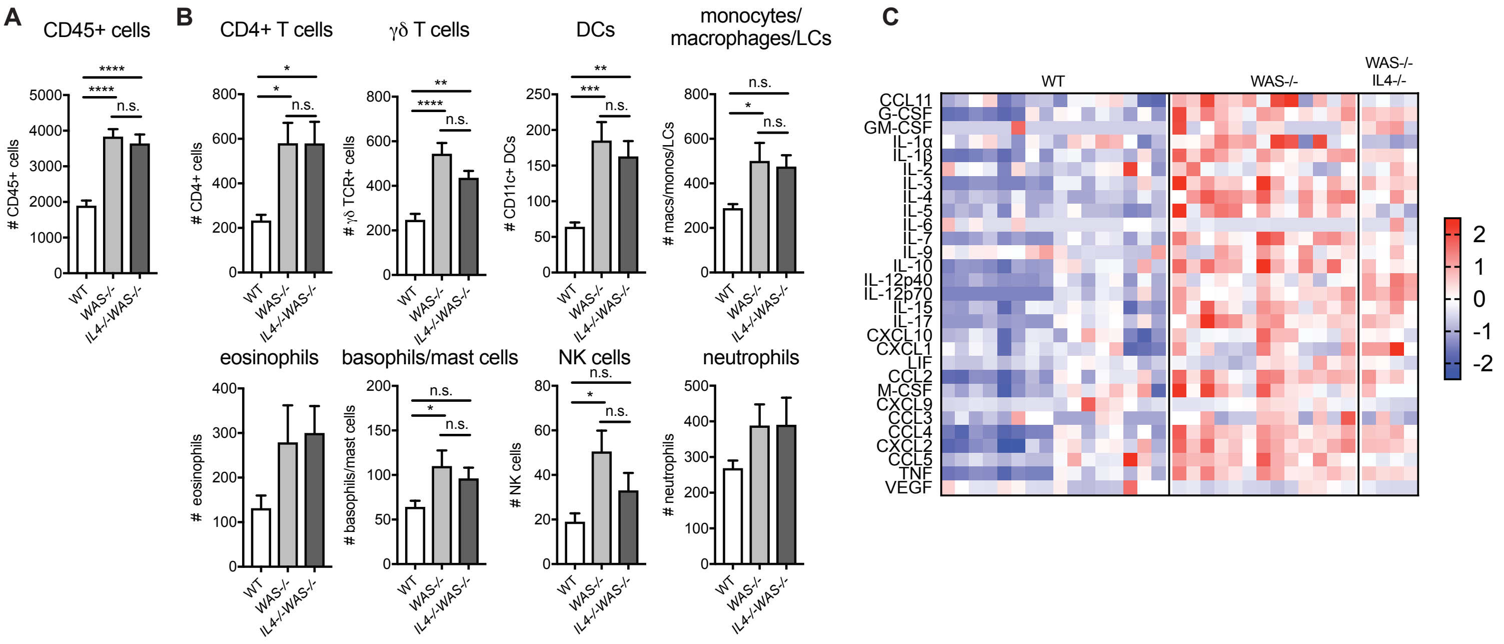

Supplemental Figure 6: Absence of IL-4 does not alleviate skin inflammation in mice lacking WASp. (A) Total CD45+ cells in ear skin of WT, WAS -/- , and IL4 -/- WAS -/- animals at eight weeks of age, measured by flow cytometry. (B) Number of individual immune cell subsets in ear skin. (C) Heat map of cytokines and chemokines in the skin, measured by Luminex assay and displayed as z-scores. n=3-5/genotype, 3 independent experiments. *p<.05, **p<.01, ***<.001,****p<.0001, One-way ANOVA with Tukey's post-test.

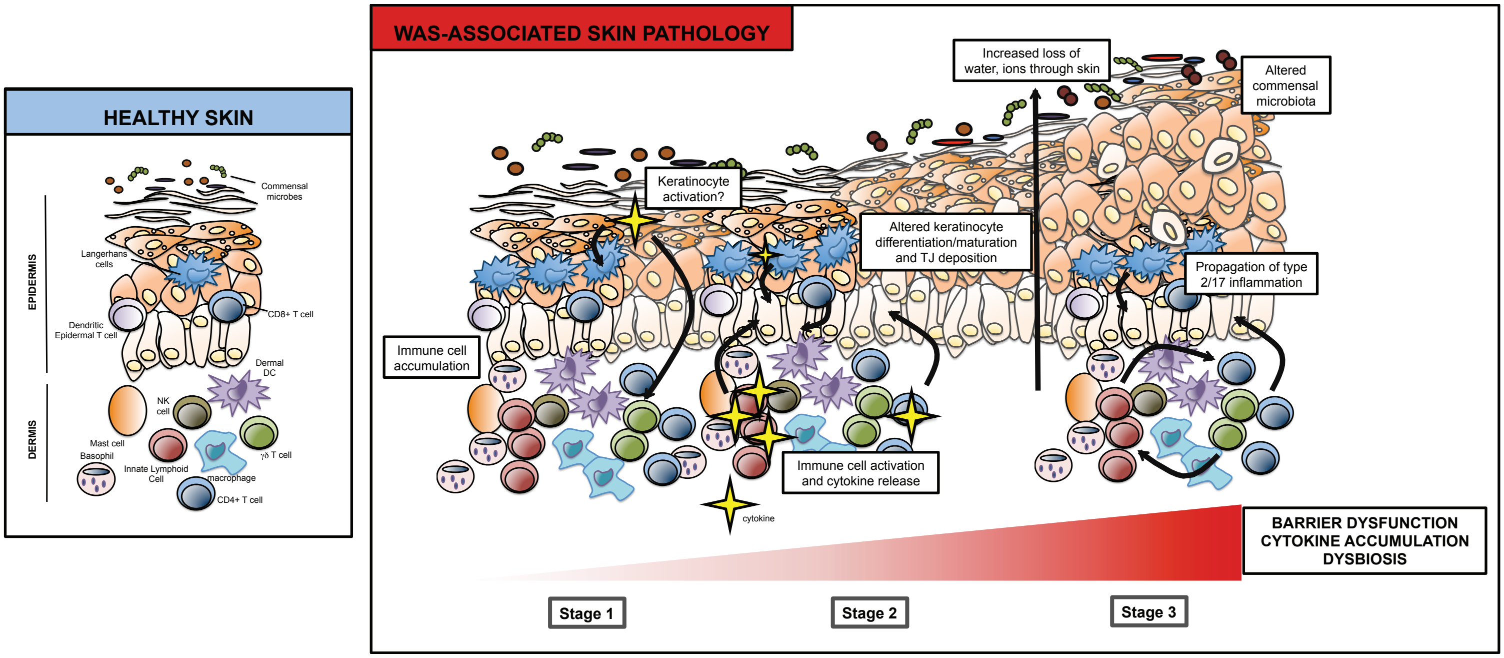

Supplemental Figure 7: Model of the development of WAS-associated skin pathology. Example of healthy skin architecture (left) for reference. In the first stage of WAS-associated skin pathology, there is an increase in the number of immune cells accumulated in WASp-deficient skin by one week of age. A trigger, perhaps from keratinocyte activation and subsequent alarmin release, activates the immune cells in the skin, leading to pro-inflammatory cytokine and chemokine release in stage 2, by four weeks of age. Cytokine accumulation in the skin results in aberrant keratinocyte differentiation and maturation and reduces tight junctional protein expression. This leads to reduction in skin physical barrier function and changes in the ecological niches present on the skin surface. Stage 3, developing by eight weeks of age, is characterized by dysbiosis due to altered skin niches and chronic inflammation, which is dominated by type 2 and 17 cytokines and chemokines, including IL-4, IL-5, IL-17, IL-22, IL-23, and CCL17.

Supplemental Table 1: Analysis of immune cell subsets by flow cytometry

Cell type	Phenotypic Markers*
Basophil/mast cell	CD45+CD49b+FcεR1+SSChi
CD4+ T cell	CD45+CD4+
CD4+ T regulatory cell	CD45+CD4+CD25+Foxp3+
CD8+ T cell	CD45+CD8β+
Dendritic cell	CD45+CD11b-CD11c+
Eosinophil	CD45+CD11b+SiglecF+SSChi
γδ T cell	CD45+γδ+CD3+
Group 2 innate lymphoid cell	CD45+Lin-CD127+GATA3+ST2+ICOS+**
Group 3 innate lymphoid cell	CD45+Lin-CD127+RORγt+NKp46+/-**
Monocyte/macrophage/langerhans cell	CD45+Ly6Glo/negCD11b+CD11c+/-
Neutrophil	CD45+CD11bhiLy6Ghi
NK cell	CD45+CD11bintCD49b+

* All cells gated first on live/singlets
**Lineage dump channel included: CD3, CD4, CD5, CD8β, CD11b, CD11c, B220, FcεR1, SiglecF, Gr-1, TER-119, and γδ TCR

Supplemental Table 2: Concentrations of cytokines/chemokines in WT and WAS-/- skin.

	WT mean	WT SEM	WAS-/- mean	WAS-/- SEM	p value	Significance	Fold change
IL-5	0.5455	0.2635	5.429	1.27	0.0006	***	9.952
IL-17	1.151	0.4538	7.985	2.326	0.0031	**	6.937
IL-4	0.1213	0.05696	0.7075	0.2733	0.0002	***	5.833
IL-22	0.6609	0.1763	3.653	1.057	0.0007	***	5.527
TNF	0.9373	0.2884	5.094	1.009	0.0001	***	5.435
IL-7	1.925	0.7688	10.106	3.705	0.027	*	5.250
LT-α	47487	7876	247260	59695	<.0001	****	5.207
CCL17	20.55	4.819	99.53	24.79	0.0011	**	4.843
IL-23	1397	200.4	6316	1785	<.0001	****	4.521
CCL4	5.499	2.568	17.01	4.443	0.0468	*	3.093
IL-12p70	14.69	2.999	45.29	10.05	0.004	**	3.083
IL-27	260.8	44.21	796.5	115.3	0.0007	***	3.054
CX3CL1	1324	183.5	4029	874.7	0.0003	***	3.043
IL-31	199.6	38.35	598	117.5	0.001	**	2.996
CXCL9	8.471	2.391	22.92	5.016	0.0138	*	2.706
CCL12	42.35	6.238	103.6	16.11	0.0019	**	2.446
IL-28b	87.53	15.04	201.3	16.14	<.0001	****	2.300
IL-11	391.5	69.93	880.9	154	0.007	**	2.250
CCL2	29.97	9.171	66.52	13.26	0.0466	*	2.220
IL-1β	27.77	27.77	58.27	58.27	0.0379	*	2.098
IL-3	3.15	0	6.44	1.797	0.007	**	2.044
CCL20	43.9	3.719	89.57	6.962	<.0001	****	2.040
IL-10	63.93	8.1	125.5	22.72	0.018	*	1.963
TIMP	401	92.49	774.6	163.1	0.0499	*	1.932
IL-20	97.25	5.669	150.8	14	0.0019	**	1.551
CXCL2	155.3	42.35	226.4	30.42	0.0499	*	1.458
CCL19	1754	199.2	2531	361.5	0.0379	*	1.443
CCL11	82.09	1.645	96.57	5.601	0.0186	*	1.176
CCL21	13368	4207	21298	4059	0.1537	n.s.	
CCL22	78.44	9.437	122.6	14.57	0.1304	n.s.	
CCL3	11.8	6.074	9.501	3.178	0.8477	n.s.	
CCL5	0	0	43.03	28.54	0.2	n.s.	
CD40L	506.8	111.3	837.1	244.3	0.7966	n.s.	
CXCL1	22.43	3.145	26.74	3.72	0.2921	n.s.	
CXCL10	11.3	2.651	16.07	3.018	0.342	n.s.	
G-CSF	15.12	3.974	18.83	2.865	0.186	n.s.	
GM-CSF	3.558	3.558	23.92	10.21	0.1282	n.s.	
IFN-γ	2.823	1.893	1.954	1.088	>.9999	n.s.	
IFNβ1	1384	195.1	2162	354	0.062	n.s.	
IL-12p40	2.594	1.06	9.23	3.591	0.1326	n.s.	
IL-15	24.61	6.239	48.13	13.06	0.1949	n.s.	
IL-16	353.7	170.6	424.2	193.2	0.2786	n.s.	
IL-1α	1909	343.5	1908	241.5	0.6454	n.s.	
IL-2	25.34	1.481	27.56	1.709	0.2894	n.s.	
IL-21	3.1	1.272	2.478	0.7125	0.934	n.s.	
IL-33	1795	305.4	1516	158.7	0.6522	n.s.	
IL-6	3.909	1.514	4.558	1.467	0.7417	n.s.	
IL-9	3501	394.1	3239	474.4	0.9591	n.s.	
LIF	1.783	0.5254	1.779	0.2573	0.5211	n.s.	
M-CSF	11.81	1.728	16.73	3.44	0.2671	n.s.	
VEGF	2.96	0.5808	3.174	0.8794	0.9009	n.s.	
CXCL5	0	0	0	0			
IL-13	0	0	0	0			

Statistics by Mann Whitney U test: *p<0.05, **p<0.01, ***p<0.001, ****p<0.0001

Supplemental Table 3: Concentrations of cytokines/chemokines in WAS-/- and Rag2-/-WAS-/- skin.

	WT mean	WT SEM	WAS-/- mean	WAS-/- SEM	Rag2-/-WAS-/- mean	Rag2-/-WAS-/- SEM	p value WAS-/- to Rag2-/-WAS-/-	Significance
**IL-5**	1.147	0.2221	4.172	0.4242	2.063	0.4235	0.0021	**
**IL-7**	5.274	1.249	25.2	2.577	13.35	3.831	0.0262	*
**IL-4**	0.1613	0.0428	1.348	0.1315	0.3988	0.09637	0.0003	***
**IL-10**	65.83	8.102	137.6	8.595	84.18	11.41	0.0127	*
**IL-12p70**	15.8	5.197	68.4	7.401	36.12	6.432	0.0211	*
**IL-17**	1.694	0.4866	7.535	0.7615	3.621	0.8515	0.0151	*
**G-CSF**	21.45	2.997	46.99	3.044	44.24	7.753	0.9883	n.s.
**IL-1β**	35.34	5.051	87.27	5.093	57.75	8.603	0.2029	n.s.
**IL-3**	2.306	0.5912	8.754	0.8627	5.948	1.504	0.369	n.s.
**CXCL10**	11.69	2.734	16.22	1.321	15.13	0.5322	0.994	n.s.
**CXCL1**	11.21	1.088	15.11	1.428	16.05	2.496	0.9907	n.s.
**LIF**	2.995	0.7299	3.125	0.3293	2.556	0.3055	0.9393	n.s.
**CCL2**	77.1	6.803	136.1	6.134	105.2	12.08	0.1803	n.s.
**CXCL9**	50.18	14.21	37.17	3.234	31.08	2.895	0.9931	n.s.
**CCL3**	6.178	2.582	9.966	2.153	5.793	1.682	0.7091	n.s.
**CCL4**	13.68	2.761	39.67	1.953	26.75	3.091	0.0538	n.s.
**CXCL2**	269.3	25.95	444.9	16.06	355.8	22.57	0.1991	n.s.
**CCL5**	1.562	0.1862	2.342	0.1213	1.789	0.167	0.472	n.s.
**TNF**	8.838	1.285	20.91	1.139	15.48	2.302	0.2051	n.s.
**IL-1α**	2165	73.88	2499	118.7	2316	116	0.6694	n.s.
**CCL11**	148.6	12.81	246.8	18.75	228.8	22.69	0.9388	n.s.
**GM-CSF**	1.228	1.228	7.452	2.603	2.074	2.074	0.4327	n.s.
**IL-2**	7.488	0.7372	7.401	0.492	7.373	0.3942	>.9999	n.s.
**IL-6**	1.056	0.545	0.1038	0.07033	0.2238	0.2112	0.9998	n.s.
**IL-9**	2064	93.86	2272	116.3	2384	106.1	0.9234	n.s.
**IL-12p40**	6.242	1.813	18.38	1.835	21.24	3.379	0.9042	n.s.
**IL-15**	60.89	8.61	135.8	10.58	98.37	13.41	0.2847	n.s.
**M-CSF**	12.19	1.403	19.62	1.653	15.45	2.024	0.5813	n.s.
**VEGF**	2.971	0.581	1.912	0.1575	2.06	0.2486	0.9972	n.s.
**IFN-γ**	0	0	0	0	0	0		
**CXCL5**	0	0	0	0	0	0		
**IL-13**	0	0	0	0	0	0		

Statistics by One-way ANOVA with Tukey's post-test: *$p<0.05$, **$p<0.01$, ***$p<0.001$.
